# Poly[bis­(μ_3_-acetato-κ^4^ *O*,*O*′:*O*:*O*′)bis­(μ_2_-acetato-κ^3^ *O*,*O*′:*O*)(μ_2_-2,5-dimethyl­benzene-1,4-diol-κ^2^ *O*:*O*′)dilead(II)]

**DOI:** 10.1107/S1600536808030535

**Published:** 2008-09-27

**Authors:** Krzysztof Lyczko, Joanna Bak

**Affiliations:** a Institute of Nuclear Chemistry and Technology, Dorodna 16, 03-195 Warsaw, Poland; b Warsaw University, Pasteura 1, 02-093 Warsaw, Poland

## Abstract

The title compound, [Pb_2_(C_2_H_3_O_2_)_4_(C_8_H_10_O_2_)]_*n*_, has a polymeric structure, with acetatolead(II) chains and 2,5-dimethyl­benzene-1,4-diol mol­ecules forming bridges between two Pb^II^ ions from neighbouring chains. Each Pb^II^ centre is surrounded by eight O atoms; four belong to bidentate acetate ions, three to neighbouring bridging acetate groups and one to the 2,5-dimethyl­benzene-1,4-diol mol­ecule. The Pb^II^ ions are chelated symmetrically and asymmetrically by acetate ligands. The coordination environment of the Pb^II^ ion can be described as a hemidirected Pb^II^O_6_ core with the empty space around the metal ion filled by the stereochemically active 6*s* ^2^ electron pair and two longer Pb—O contacts. The Pb—O distances are in the range of 2.355 (3)–2.994 (3) Å. Additionally, the crystal structure is stabilized by O—H⋯O hydrogen bonds.

## Related literature

Other crystal structures containing polymeric lead(II) acetate were reported by: Rajaram & Rao (1982 ▶); Bryant *et al.* (1984 ▶); Harrowfield *et al.* (1996 ▶). Van der Waals radii of lead(II) and oxygen were presented by Bondi (1964 ▶). The synthesis of the title compound was carried out similarly to the method used for obtaining the bis­(2,4-penta­nedionato)lead(II) complex described by Lyczko *et al.* (2006 ▶). Hemi- and holodirected geometries of lead(II) complexes and the role of the 6*s* ^2^ lone electron pair of the lead(II) ion were discussed by Shimoni-Livny *et al.* (1998 ▶).
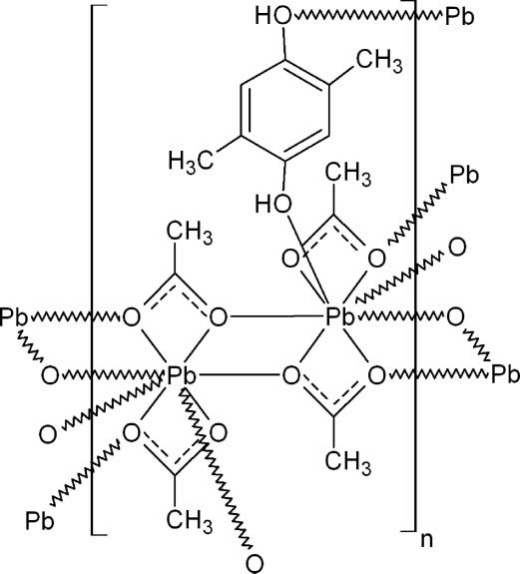


## Experimental

### 

#### Crystal data

[Pb_2_(C_2_H_3_O_2_)_4_(C_8_H_10_O_2_)]
*M* *_r_* = 788.72
Triclinic, 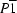

*a* = 7.4905 (11) Å
*b* = 7.5526 (10) Å
*c* = 10.2522 (15) Å
α = 93.043 (11)°
β = 100.787 (12)°
γ = 116.377 (14)°
*V* = 504.28 (12) Å^3^
*Z* = 1
Mo *K*α radiation
μ = 16.72 mm^−1^
*T* = 100 (2) K
0.35 × 0.26 × 0.14 mm

#### Data collection

Kuma KM-4 four-circle CCD diffractometer
Absorption correction: analytical (*CrysAlis RED*; Oxford Diffraction, 2005 ▶) *T*_min_ = 0.040, *T*_max_ = 0.227
9065 measured reflections
2432 independent reflections
2317 reflections with *I* > 2σ(*I*)
*R*_int_ = 0.041

#### Refinement

*R*[*F* ^2^ > 2σ(*F* ^2^)] = 0.017
*wR*(*F* ^2^) = 0.043
*S* = 1.07
2432 reflections
132 parameters
H-atom parameters constrained
Δρ_max_ = 1.30 e Å^−3^
Δρ_min_ = −1.67 e Å^−3^

### 

Data collection: *CrysAlis CCD* (Oxford Diffraction, 2005 ▶); cell refinement: *CrysAlis RED* (Oxford Diffraction, 2005 ▶); data reduction: *CrysAlis RED*; program(s) used to solve structure: *SHELXS97* (Sheldrick, 2008 ▶); program(s) used to refine structure: *SHELXL97* (Sheldrick, 2008 ▶); molecular graphics: *Mercury* (Macrae *et al.*, 2006 ▶); software used to prepare material for publication: *SHELXL97*.

## Supplementary Material

Crystal structure: contains datablocks global, I. DOI: 10.1107/S1600536808030535/nc2114sup1.cif

Structure factors: contains datablocks I. DOI: 10.1107/S1600536808030535/nc2114Isup2.hkl

Additional supplementary materials:  crystallographic information; 3D view; checkCIF report

## Figures and Tables

**Table 1:** Selected bond lengths (Å)

Pb1—O3	2.355 (3)
Pb1—O1	2.493 (3)
Pb1—O2	2.499 (3)
Pb1—O1^i^	2.618 (2)
Pb1—O4	2.700 (3)
Pb1—O2^ii^	2.767 (3)
Pb1—O5	2.993 (3)
Pb1—O4^iii^	2.994 (3)

Symmetry codes: (i) 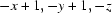; (ii) 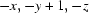; (iii) 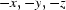.

**Table 2:** Hydrogen-bond geometry (Å, °)

*D*—H⋯*A*	*D*—H	H⋯*A*	*D*⋯*A*	*D*—H⋯*A*
O5—H5⋯O3^i^	0.84	1.87	2.668 (4)	159

Symmetry code: (i) 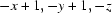.

**Fig. 1.**
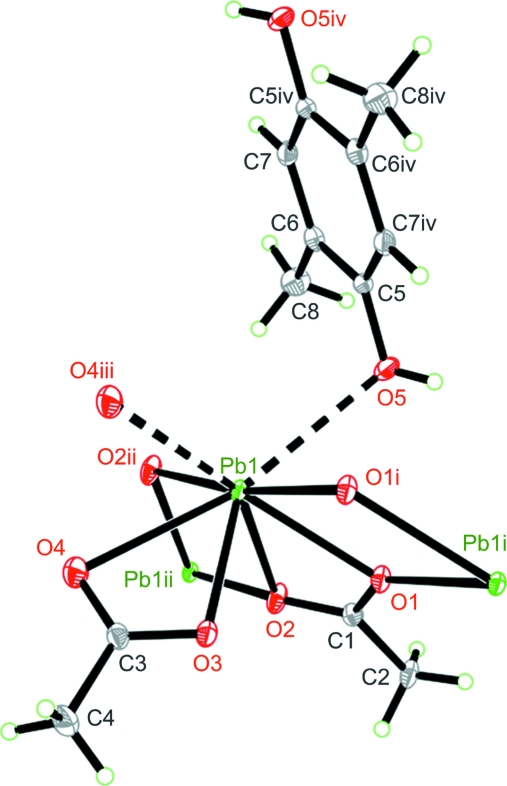
A fragment of the polymeric structure of the title compound with labelling showing the environment of the lead atom. Displacement ellipsoids of non-hydrogen atoms are drawn at the 50% probability level. [Symmetry codes: (i) -x + 1, -y + 1, -z; (ii) -x, -y + 1, -z; (iii) -x, -y, -z; (iv) x + 1, -y + 1, -z + 1].

**Fig. 2.**
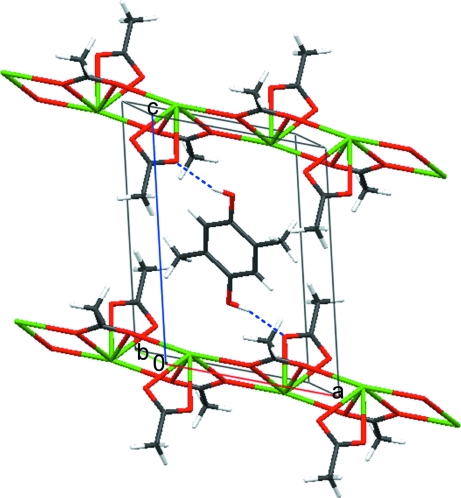
A packing diagram of the title compound. Hydrogen bonding is shown as blue dashed lines.

## supplementary crystallographic information

### Comment

The aim of this work was to synthesize the complex between Pb^II^ ions and 3-methyl-2,4-pentanedione. Unexpectedly, under the reaction conditions (see *Experimental*), instead of the bis(3-methyl-2,4-pentanedionato)lead(II) complex, the title compound was obtained.

The structure of this compound consists of polymeric chains of lead(II) acetate linked *via* 2,5-dimethylbenzene-1,4-diol molecules, each bridging two Pb^II^ ions from neighbouring chains. As shown in Fig. 1 the title compound contains eight-coordinated Pb^II^ ions. Each Pb^II^ centre binds strongly four O atoms from bidentate acetate ions in a nearly pyramidal configuration with Pb—O bond lengths in the range of 2.355 (3)–2.700 (3) Å, and additionally two O atoms from neighbouring bridging acetate groups of the same chain at 2.618 (2) and 2.766 (3) Å. The Pb^II^ ions are chelated symmetrically [Pb—O = 2.493 (3) and 2.499 (3) Å] and asymmetrically [2.355 (3) and 2.700 (3) Å] by acetato ligands. The coordination sphere of the metal ion is completed by two additional O atoms at distances of about 2.99 Å, the first from the acetate group of the neighbouring chain and the second from the 2,5-dimethylbenzene-1,4-diol molecule. These two contacts are much longer than the Pb—O bonds in the chelate but shorter than the sum of the van der Waals radii of lead(II) and oxygen (3.44 Å; Bondi, 1964) indicating some weak interactions. The crystal structure is also stabilized by O—H···O hydrogen bonds. The OH groups of the 2,5-dimethylbenzene-1,4-diol molecule form hydrogen bonds with a H···O distance of 1.87 Å to O atoms from neighbouring CH_3_COO^-^ ions (Fig. 2). The resulting coordination sphere of the Pb^II^ ion in the title compound has a hemidirected geometry (Shimoni-Livny *et al.*, 1998). It contains the Pb^II^O_6_ core with the empty space at one side of the metal ion filled by stereochemically active electron pair and two longer Pb—O contacts of 2.993 (3) and 2.994 (3) Å. The 6*s*^2^ electron pair is located opposite to the shortest Pb—O bond length [2.355 (3) Å] and between two longest Pb—O contacts of about 2.99 Å.

Identical polymeric acetato-lead(II) chains are present in the lead(II) acetate trihydrate structure, where the metal ions are nine-coordinated (Pb^II^O_9_) with two bidentate acetate groups, three coordinated water molecules and two bridging distances to O atoms from the neighbouring acetate groups (Rajaram & Rao, 1982; Bryant *et al.*, 1984). In another polymeric structure of lead(II) acetate the Pb^II^ ions are eight-coordinated with the Pb^II^O_6_N_2_ core containing in addition two N atoms from two 2-pyridylamine molecules (Harrowfield *et al.*, 1996).

### Experimental

The title compound was obtained unintentionally in an attempt to synthesize single crystals of the bis(3-methyl-2,4-pentanedionato)lead(II) complex. Small pieces of lead foil (0.26 g) were heated (~50 °C) for a few weeks in a mixture of 3-methyl-2,4-pentanedione (2 ml) and toluene (2 ml) under reflux. The synthesis was carried out similarly to that used for the preparation of crystals of the bis(2,4-pentanedionato)lead(II) complex (Lyczko *et al.*, 2006). After heating the reaction mixture was stored for a few months until it converted into an orange–brown solid. Colourless crystals of the title compound were separated from the reaction mixture. Analysis, calculated for C_16_H_22_O_10_Pb_2_: C 24.36, H 2.81%; found: C 24.14, H 2.88%.

### Refinement

H atoms were placed in calculated positions (O—H H atoms allowed to rotate but not to tip) with C—H = 0.98 Å (CH_3_), C—H = 0.95 Å (aromatic H atoms), O—H = 0.84 Å and were refined isotropic with *U*_iso_(H) = 1.5*U*_eq_(C, O) and *U*_iso_(H) = 1.2*U*_eq_(C) for aromatic H atoms using a riding model.

### Crystal data

**Table:**

[Pb_2_(C_2_H_3_O_2_)_4_(C_8_H_10_O_2_)]	*Z* = 1
*M**_r_* = 788.72	*F*(000) = 362
Triclinic, *P*1	*D*_x_ = 2.597 Mg m^−^^3^
Hall symbol: -P 1	Mo *K*α radiation, λ = 0.71073 Å
*a* = 7.4905 (11) Å	Cell parameters from 9065 reflections
*b* = 7.5526 (10) Å	θ = 3.0–28.7°
*c* = 10.2522 (15) Å	µ = 16.72 mm^−^^1^
α = 93.043 (11)°	*T* = 100 K
β = 100.787 (12)°	Prismatic, colourless
γ = 116.377 (14)°	0.35 × 0.26 × 0.14 mm
*V* = 504.28 (12) Å^3^	

### Data collection

**Table:**

Kuma KM-4 four-circle CCD diffractometer	2432 independent reflections
Radiation source: fine-focus sealed tube	2317 reflections with *I* > 2σ(*I*)
graphite	*R*_int_ = 0.041
Detector resolution: 8.6479 pixels mm^-1^	θ_max_ = 28.7°, θ_min_ = 3.1°
ω scans	*h* = −9→9
Absorption correction: analytical (CrysAlis RED; Oxford Diffraction, 2005)	*k* = −10→10
*T*_min_ = 0.040, *T*_max_ = 0.227	*l* = −13→13
9065 measured reflections	

### Refinement

**Table:**

Refinement on *F*^2^	Secondary atom site location: difference Fourier map
Least-squares matrix: full	Hydrogen site location: inferred from neighbouring sites
*R*[*F*^2^ > 2σ(*F*^2^)] = 0.017	H-atom parameters constrained
*wR*(*F*^2^) = 0.043	*w* = 1/[σ^2^(*F*_o_^2^) + (0.0223*P*)^2^ + 0.6288*P*] where *P* = (*F*_o_^2^ + 2*F*_c_^2^)/3
*S* = 1.07	(Δ/σ)_max_ = 0.001
2432 reflections	Δρ_max_ = 1.30 e Å^−^^3^
132 parameters	Δρ_min_ = −1.67 e Å^−^^3^
0 restraints	Extinction correction: SHELXL97 (Sheldrick, 2008), Fc^*^=kFc[1+0.001xFc^2^λ^3^/sin(2θ)]^-1/4^
Primary atom site location: structure-invariant direct methods	Extinction coefficient: 0.0077 (5)

### Special details

**Table:**

Geometry. All e.s.d.'s (except the e.s.d. in the dihedral angle between two l.s. planes) are estimated using the full covariance matrix. The cell e.s.d.'s are taken into account individually in the estimation of e.s.d.'s in distances, angles and torsion angles; correlations between e.s.d.'s in cell parameters are only used when they are defined by crystal symmetry. An approximate (isotropic) treatment of cell e.s.d.'s is used for estimating e.s.d.'s involving l.s. planes.
Refinement. Refinement of *F*^2^ against ALL reflections. The weighted *R*-factor *wR* and goodness of fit *S* are based on *F*^2^, conventional *R*-factors *R* are based on *F*, with *F* set to zero for negative *F*^2^. The threshold expression of *F*^2^ > σ(*F*^2^) is used only for calculating *R*-factors(gt) *etc*. and is not relevant to the choice of reflections for refinement. *R*-factors based on *F*^2^ are statistically about twice as large as those based on *F*, and *R*- factors based on ALL data will be even larger.

### Fractional atomic coordinates and isotropic or equivalent isotropic displacement parameters (Å^2^)

**Table:**

	*x*	*y*	*z*	*U*_iso_*/*U*_eq_	
Pb1	0.205617 (17)	0.362095 (17)	0.046792 (11)	0.00910 (6)	
O1	0.4490 (4)	0.6463 (4)	−0.0457 (2)	0.0121 (5)	
O2	0.1324 (4)	0.6005 (4)	−0.0858 (3)	0.0154 (5)	
O3	0.1663 (4)	0.2264 (4)	−0.1751 (2)	0.0148 (5)	
O4	−0.1191 (4)	0.0447 (4)	−0.1145 (3)	0.0214 (6)	
C1	0.3154 (5)	0.6990 (5)	−0.0954 (3)	0.0111 (6)	
C2	0.3723 (6)	0.8784 (6)	−0.1662 (4)	0.0155 (7)	
H2A	0.5180	0.9365	−0.1668	0.023*	
H2B	0.3466	0.9780	−0.1192	0.023*	
H2C	0.2896	0.8380	−0.2589	0.023*	
C3	−0.0132 (5)	0.0788 (6)	−0.1992 (4)	0.0135 (7)	
C4	−0.0903 (6)	−0.0587 (6)	−0.3316 (4)	0.0212 (8)	
H4A	−0.0509	−0.1658	−0.3216	0.032*	
H4B	−0.0301	0.0174	−0.4002	0.032*	
H4C	−0.2400	−0.1172	−0.3588	0.032*	
C5	0.5070 (5)	0.5941 (5)	0.3871 (3)	0.0124 (7)	
O5	0.5039 (4)	0.6836 (4)	0.2743 (3)	0.0166 (5)	
H5	0.6216	0.7356	0.2591	0.025*	
C6	0.3672 (5)	0.5861 (6)	0.4637 (4)	0.0129 (7)	
C7	0.3647 (5)	0.4909 (5)	0.5766 (3)	0.0133 (7)	
H7	0.2720	0.4841	0.6303	0.016*	
C8	0.2300 (6)	0.6797 (7)	0.4230 (4)	0.0207 (8)	
H8A	0.1526	0.6732	0.4914	0.031*	
H8B	0.1344	0.6074	0.3366	0.031*	
H8C	0.3133	0.8199	0.4145	0.031*	

### Atomic displacement parameters (Å^2^)

**Table:**

	*U*^11^	*U*^22^	*U*^33^	*U*^12^	*U*^13^	*U*^23^
Pb1	0.00762 (8)	0.00740 (8)	0.01117 (8)	0.00219 (6)	0.00281 (5)	0.00244 (5)
O1	0.0087 (12)	0.0098 (12)	0.0179 (12)	0.0040 (10)	0.0032 (9)	0.0049 (10)
O2	0.0082 (12)	0.0150 (13)	0.0246 (14)	0.0054 (11)	0.0062 (10)	0.0077 (11)
O3	0.0130 (12)	0.0112 (12)	0.0157 (12)	0.0012 (11)	0.0048 (9)	0.0017 (10)
O4	0.0123 (13)	0.0247 (16)	0.0203 (14)	0.0021 (12)	0.0053 (10)	0.0018 (12)
C1	0.0119 (16)	0.0087 (16)	0.0123 (16)	0.0048 (14)	0.0025 (12)	0.0006 (13)
C2	0.0098 (17)	0.0120 (18)	0.0240 (19)	0.0034 (15)	0.0051 (14)	0.0091 (15)
C3	0.0107 (17)	0.0115 (17)	0.0151 (16)	0.0037 (15)	−0.0002 (13)	0.0028 (14)
C4	0.023 (2)	0.0139 (19)	0.0179 (18)	0.0035 (17)	0.0004 (15)	−0.0014 (15)
C5	0.0120 (16)	0.0105 (17)	0.0090 (15)	0.0010 (14)	0.0011 (12)	0.0005 (13)
O5	0.0146 (13)	0.0202 (14)	0.0150 (12)	0.0062 (12)	0.0061 (10)	0.0089 (11)
C6	0.0110 (17)	0.0105 (16)	0.0145 (16)	0.0036 (14)	0.0013 (12)	−0.0007 (13)
C7	0.0109 (16)	0.0128 (17)	0.0133 (16)	0.0029 (14)	0.0038 (12)	0.0001 (14)
C8	0.0190 (19)	0.025 (2)	0.0224 (19)	0.0138 (18)	0.0055 (15)	0.0065 (16)

### Geometric parameters (Å, °)

**Table:**

Pb1—O3	2.355 (3)	C2—H2C	0.9800
Pb1—O1	2.493 (3)	C3—C4	1.509 (5)
Pb1—O2	2.499 (3)	C4—H4A	0.9800
Pb1—O1^i^	2.618 (2)	C4—H4B	0.9800
Pb1—O4	2.700 (3)	C4—H4C	0.9800
Pb1—O2^ii^	2.767 (3)	C5—O5	1.372 (4)
Pb1—O5	2.993 (3)	C5—C7^iv^	1.384 (5)
Pb1—O4^iii^	2.994 (3)	C5—C6	1.405 (5)
O1—C1	1.267 (4)	O5—H5	0.8400
O1—Pb1^i^	2.618 (2)	C6—C7	1.393 (5)
O2—C1	1.263 (4)	C6—C8	1.499 (5)
O3—C3	1.275 (4)	C7—C5^iv^	1.384 (5)
O4—C3	1.245 (4)	C7—H7	0.9500
C1—C2	1.500 (5)	C8—H8A	0.9800
C2—H2A	0.9800	C8—H8B	0.9800
C2—H2B	0.9800	C8—H8C	0.9800
			
O3—Pb1—O1	75.58 (9)	O4—C3—O3	121.2 (3)
O3—Pb1—O2	77.94 (9)	O4—C3—C4	120.8 (3)
O1—Pb1—O2	52.08 (8)	O3—C3—C4	118.0 (3)
O3—Pb1—O1^i^	75.04 (8)	C3—C4—H4A	109.5
O1—Pb1—O1^i^	66.16 (9)	C3—C4—H4B	109.5
O2—Pb1—O1^i^	116.77 (8)	H4A—C4—H4B	109.5
O3—Pb1—O4	50.93 (8)	C3—C4—H4C	109.5
O1—Pb1—O4	121.84 (8)	H4A—C4—H4C	109.5
O2—Pb1—O4	91.50 (9)	H4B—C4—H4C	109.5
O1^i^—Pb1—O4	111.98 (8)	O5—C5—C7^iv^	122.7 (3)
C1—O1—Pb1	94.0 (2)	O5—C5—C6	116.7 (3)
C1—O1—Pb1^i^	149.3 (2)	C7^iv^—C5—C6	120.6 (3)
Pb1—O1—Pb1^i^	113.84 (9)	C5—O5—H5	109.5
C1—O2—Pb1	93.8 (2)	C7—C6—C5	117.4 (3)
C3—O3—Pb1	101.7 (2)	C7—C6—C8	122.9 (3)
C3—O4—Pb1	86.2 (2)	C5—C6—C8	119.8 (3)
O2—C1—O1	120.1 (3)	C5^iv^—C7—C6	122.0 (3)
O2—C1—C2	119.4 (3)	C5^iv^—C7—H7	119.0
O1—C1—C2	120.6 (3)	C6—C7—H7	119.0
C1—C2—H2A	109.5	C6—C8—H8A	109.5
C1—C2—H2B	109.5	C6—C8—H8B	109.5
H2A—C2—H2B	109.5	H8A—C8—H8B	109.5
C1—C2—H2C	109.5	C6—C8—H8C	109.5
H2A—C2—H2C	109.5	H8A—C8—H8C	109.5
H2B—C2—H2C	109.5	H8B—C8—H8C	109.5
			
O3—Pb1—O1—C1	86.8 (2)	O2—Pb1—O4—C3	74.2 (2)
O2—Pb1—O1—C1	1.04 (19)	O1^i^—Pb1—O4—C3	−45.6 (2)
O1^i^—Pb1—O1—C1	166.6 (3)	Pb1—O2—C1—O1	1.9 (3)
O4—Pb1—O1—C1	64.6 (2)	Pb1—O2—C1—C2	−178.1 (3)
O3—Pb1—O1—Pb1^i^	−79.76 (11)	Pb1—O1—C1—O2	−1.9 (3)
O2—Pb1—O1—Pb1^i^	−165.57 (16)	Pb1^i^—O1—C1—O2	153.6 (3)
O1^i^—Pb1—O1—Pb1^i^	0.0	Pb1—O1—C1—C2	178.1 (3)
O4—Pb1—O1—Pb1^i^	−101.97 (11)	Pb1^i^—O1—C1—C2	−26.4 (6)
O3—Pb1—O2—C1	−82.0 (2)	Pb1—O4—C3—O3	−1.8 (3)
O1—Pb1—O2—C1	−1.05 (19)	Pb1—O4—C3—C4	175.7 (3)
O1^i^—Pb1—O2—C1	−15.8 (2)	Pb1—O3—C3—O4	2.1 (4)
O4—Pb1—O2—C1	−131.5 (2)	Pb1—O3—C3—C4	−175.4 (3)
O1—Pb1—O3—C3	−156.6 (2)	O5—C5—C6—C7	178.3 (3)
O2—Pb1—O3—C3	−103.1 (2)	C7^iv^—C5—C6—C7	0.3 (6)
O1^i^—Pb1—O3—C3	134.7 (2)	O5—C5—C6—C8	−2.3 (5)
O4—Pb1—O3—C3	−1.1 (2)	C7^iv^—C5—C6—C8	179.8 (4)
O3—Pb1—O4—C3	1.1 (2)	C5—C6—C7—C5^iv^	−0.3 (6)
O1—Pb1—O4—C3	29.2 (2)	C8—C6—C7—C5^iv^	−179.8 (4)

Symmetry codes: (i) −*x*+1, −*y*+1, −*z*; (ii) −*x*, −*y*+1, −*z*; (iii) −*x*, −*y*, −*z*; (iv) −*x*+1, −*y*+1, −*z*+1.

### Hydrogen-bond geometry (Å, °)

**Table:**

*D*—H···*A*	*D*—H	H···*A*	*D*···*A*	*D*—H···*A*
O5—H5···O3^i^	0.84	1.87	2.668 (4)	159.

Symmetry codes: (i) −*x*+1, −*y*+1, −*z*.
